# Exploring the Prognostic Potential of circSCORE in Patients with Relapsed/Refractory Mantle Cell Lymphoma

**DOI:** 10.3390/genes16060634

**Published:** 2025-05-25

**Authors:** Ruth Salim, Christian Winther Eskelund, Mats Jerkeman, Arne Kolstad, Riikka Räty, Christian Geisler, Martin Hutchings, Carsten Utoft Niemann, Lone Bredo Pedersen, Jonas Raaschou-Pedersen, Eileen Wedge, Juan Luis García-Rodríguez, Lasse Sommer Kristensen, Mette Dahl, Kirsten Grønbæk

**Affiliations:** 1 Department of Hematology, Copenhagen University Hospital, Rigshospitalet, 2100 Copenhagen, Denmark; ruth.salim.01@regionh.dk (R.S.); kirsten.groenbaek@regionh.dk (K.G.); 2 Biotech Research and Innovation Centre, BRIC, University of Copenhagen, 2200 Copenhagen, Denmark; 3 Department of Oncology, Skane University Hospital, SE-221 00 Lund, Sweden; 4 Department of Oncology, Division Gjøvik-Lillehammer, Innlandet Hospital Trust, 2819 Gjøvik, Norway; 5 Department of Hematology, Helsinki University Hospital, 00029 Helsinki, Finland; 6 Department of Clinical Medicine, Faculty of Health Sciences, University of Copenhagen, 2200 Copenhagen, Denmark; 7 Faculty of Social Sciences, University of Copenhagen, 1353 Copenhagen, Denmark; 8 Department of Biomedicine, Aarhus University, 8000 Aarhus, Denmark

**Keywords:** ncRNA, circRNA, circSCORE, risk score, biomarker, mantle cell lymphoma, relapse/refractory disease, NanoString

## Abstract

**Background and Objectives**: Mantle cell lymphoma (MCL) is a highly heterogenous disease, but an optimal prognostic biomarker in relapsed/refractory (R/R) MCL has not yet been established. The circular RNA-based risk score, circSCORE, was recently proposed as a promising prognosticator in newly diagnosed, younger patients with MCL. This study explores the prognostic potential of circSCORE in R/R MCL in both nodal (lymph node (LN)) and non-nodal tissues (bone marrow (BM)) and peripheral blood (PB)). **Materials and Methods**: RNA was extracted from 65 relapse samples consisting of first-relapse LN samples (*n* = 20) from patients who previously underwent first-line treatment in the MCL2 and MCL3 trials, and either BM (*n* = 34) or PB (*n* = 11) samples obtained from patients with R/R MCL included in the MCL6 trial, taken at trial baseline. Kaplan–Meier estimates, and Cox regressions were used to evaluate the association between circSCORE risk groups (high versus low) and outcomes. **Results**: Survival analyses showed significantly inferior outcomes for patients with high-risk circSCORE compared to low-risk score for both progression-free survival (PFS) (hazard ratio (HR) 1.99, *p*-value 0.0407) and overall survival (OS) (HR 2.29, *p*-value 0.0192) in the total cohort. The same tendencies were displayed when exploring the non-nodal samples only. Furthermore, circSCORE retained prognostic impact for PFS, but not OS, when adjusted for Ki67, MIPI, and *TP53* mutation status. **Conclusions**: The circRNA-based risk score, circSCORE, displayed prognostic potential in R/R MCL along with promising application in non-nodal tissues, indicating that circSCORE, if further validated, might serve as an easily obtainable biomarker in R/R MCL.

## 1. Introduction

Mantle cell lymphoma (MCL) is a B-cell non-Hodgkin lymphoma subtype characterized by biological and clinical heterogenicity and presenting with variable prognosis, response rates, and a continuous relapse pattern. At relapse, treatment is individualized, mainly based on clinical assessment, yet no optimal prognostic biomarker in relapsed/refractory (R/R) MCL has been established [1]. A commonly used prognostic tool at diagnosis is the MCL International Prognostic Index (MIPI) based on clinical variables [2], which is often incorporated with the immunohistochemical Ki67 proliferation index to add biological information in the MIPI-combined (MIPI-C) risk score [3]. However, the immunohistochemical methods on which the Ki67 marker is based are prone to intra- and interobserver variability, thereby limiting its use for clinical decision-making. When available, the *TP53* mutation status is a strong prognosticator at diagnosis [4], but it remains to be incorporated into routine diagnostic practice. Recently, a circular RNA (circRNA)-based risk score, circSCORE, showed promising prognostic performance in younger ≤65 years), newly diagnosed patients with MCL [5,6].

CircRNAs are non-coding molecules formed by an alternative back-splicing mechanism linking an upstream splice acceptor (3′ splice site) to a downstream splice donor (5′ splice site), resulting in circular formation [7]. Due to the lack of free ends, circRNAs are resistant to exonucleolytic degradation, thereby ensuring high stability and long half-lives compared to their linear counterparts [8]. In contrast to messenger RNAs (mRNAs), circRNAs are very rarely translated into peptides, but a few cases have been reported [9,10]. Rather, the prevailing consensus of circRNA functions regard direct interactions with other molecules, such as regulating microRNAs (miRNAs), mediate protein complexes by scaffolding, recruiting or retaining specific proteins in designated cellular compartments, and competing with the transcription of their host gene [11,12]. Although the majority of circRNAs are expressed at very low levels, they tend to accumulate in slow-proliferating cells and dilute in high-proliferating cells, such as cancerous cells [13]. Moreover, circRNAs display disease-specific expression patterns as well as altered expressions in many cancers, including B-cell lymphomas [14]. Studies have demonstrated that circRNAs are detectable in various tissue types, including blood, with a promising potential to serve as biomarkers with the use of less invasive methods than tumor biopsies [15,16,17,18].

The identification of circRNAs has previously been challenging, but the recently suggested circSCORE can be measured using the NanoString nCounter technology, which is an enzyme-free method suitable for the detection and accurate quantification of circRNAs [19]. Therefore, circSCORE has been suggested to represent a biologically relevant and more objective prognosticator in newly diagnosed patients with MCL. However, no studies have assessed the prognostic potential of circSCORE in R/R MCL, and it has never been evaluated solely in non-nodal tissue before. The aim of this study was to investigate the prognostic potential of the newly proposed circSCORE in patients with R/R MCL and explore circSCORE performance in non-nodal tissue.

## 2. Materials and Methods

### 2.1. Cohorts and Patient Material

A total of 65 relapse samples were available and included in this study, all originating from patients with R/R MCL who had previously been included in the Nordic MCL2 (ISRCTN87866680), MCL3 (NCT00514475) and MCL6 (NCT02460276) clinical trials. Patient and disease characteristics for the included patients in this study are presented in the Supplemental Material, Table S1. Further details on the original cohorts are described in the studies by Geisler et al., 2012 [20], Kolstad et al., 2014 [21], and Jerkeman et al., 2018 [22], respectively. In short, newly diagnosed patients enrolled in MCL2 and MCL3 all received high-dose cytarabine-containing chemoimmunotherapy and autologous stem cell therapy (ASCT), while relapse treatment varied and was given outside of the protocol (Supplemental Material, Table S2). In the MCL6 trial, patients with R/R MCL, previously treated with at least one Rituximab-containing regimen, were treated uniformly after inclusion in the trial with Ibrutinib, Lenalidomide, and Rituximab. For the MCL2 and MCL3 samples, RNA was isolated from lymph node tissue obtained at first relapse (*n* = 20), and for the MCL6 cohort from relapse samples taken from the bone marrow (BM) (*n* = 34) or peripheral blood (PB) (*n* = 11) at the baseline when BM was not available. MCL involvement in the BM samples was determined by pathological evaluation, and PB involvement was defined as a peripheral white blood cell count (WBC) exceeding the upper limit (10.7 × 10^9^/L) with a differential count of lymphocytes >5 × 10^9^/L [23].

### 2.2. RNA Isolation and circSCORE Calcutaion

RNA was isolated from all 65 samples using the *Quick*-DNA/RNA MicroPrep Plus Kit (Zymo Research, Irvine, CA, USA) according to the manufacturer’s instructions. RNA expression was carried out on the nCounter^TM^ SPRINT platform (Nanostring Technologies, Seattle, WA, USA) using 200 nanograms of RNA and applying the NanoString CodeSet design developed by Dahl et al. (2022) [5]. In short, CodeSet consists of 50 base pair (bp) capture probes and 50 bp reporter probes, designed to target regions of nucleotides overlaying the unique back-splicing junctions of selected circRNAs. Since each back-splicing junction is unique to its corresponding circRNA, this approach ensures that only circRNAs, and not any corresponding linear RNAs that might be present in the sample, are detected. * Analyses were carried out using the nSOLVER Analysis Software (version 4.0.70, NanoString Technologies).

Because circSCORE was previously developed and proposed in 2022 by Dahl et al., the NanoString CodeSet design and circSCORE calculation were performed according to the original circSCORE paper. The calculation of the circSCORE for each of the 65 patients included in this study was, therefore, based on individual (log) expression levels of the nine circRNAs that the circSCORE is based on (circANKRD17, circARID1A, circEXOC6B, circFAM13B, circNCOA2, circPNN, circRAB11FIP1, circZCCHC6, circZNF609) and their respective regression coefficients using the following formula:

circSCORE = −0.044 × log(circANKRD17) + 0.245 × log(circARID1A) + −0.247 × log(circEXOC6B) + −0.402 × log(circFAM13B) + 0.356 × log(circNCOA2) + −0.146 × log(circPNN) + −0.413 × log(circRAB11FIP1) + −0.028 × log(circZCCHC6) + −0.105 × log(circZNF609)

Also, in accordance with the original circSCORE paper, the cut-off value of −7.065 was used to stratify the patients into a high-risk group and a low-risk group. All information on circSCORE development, NanoString CodeSet design, and circSCORE calculation can be found and is described in detail in the original circSCORE paper by Dahl et al. (2022) [5].

### 2.3. High-Risk Features and Survival Analyses

In 49 of the 65 patients, data on the other high-risk features, including the MIPI score, Ki67, and *TP53* mutation status obtained at time of relapse, were all available. The *TP53* mutation analyses were previously performed and described in the studies by Eskelund et al. (2017) [4] and Jerkeman et al. (2018) [22]. Progression-free survival (PFS) and overall survival (OS) were set as endpoints for survival analyses, with the starting point for both being the date of the obtainment of the relapse sample (the first relapse sample for MCL2/3 and baseline sample for MCL6). The endpoint for PFS was the date of disease progression, treatment failure, or death of any course, while the endpoint for OS was death due to any cause. All tests were performed at a 5% significance level. All statistical analyses were carried out on the statistical software R version 4.0.3.

## 3. Results

### 3.1. Inferior Outcomes for Patients with High-Risk circSCORE Compared to Low-Risk circSCORE 

**Figure 1:**
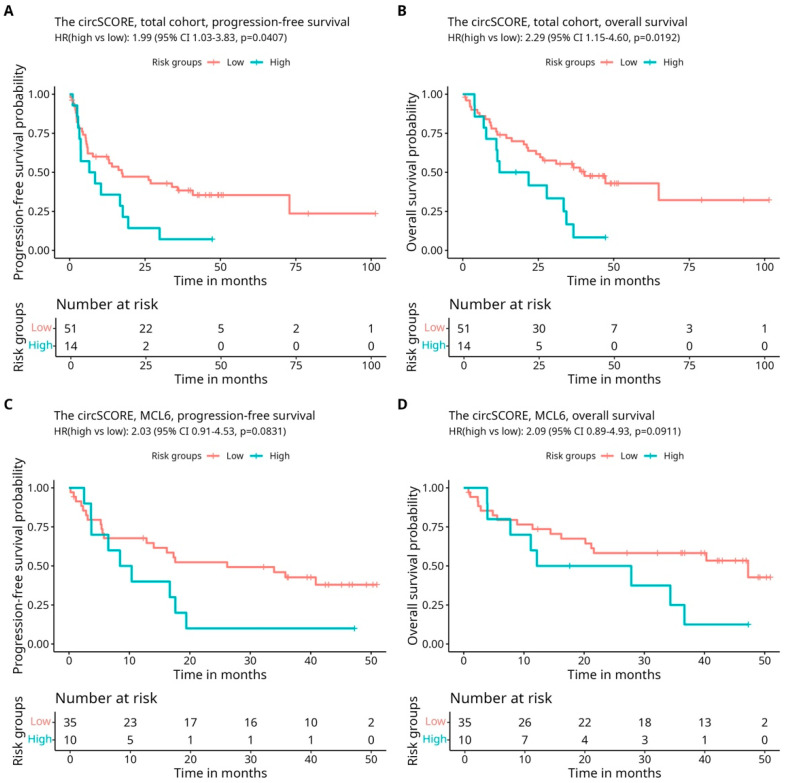
**Kaplan–Meier survival curves and Cox univariable regressions of PFS and OS stratified on circSCORE risk groups in the total cohort and in MCL6 only** Kaplan–Meier survival curves and univariable Cox regressions performed in the total cohort of MCL2, MCL3, and MCL6 (n = 65) (**A**,**B**) and in the non-nodal MCL6 cohort (n = 45) (**C**,**D**). Patients were stratified in circSCORE high- and low-risk groups, and endpoints were progression-free survival (PFS) (**A**,**C**) and overall survival (OS) (**B**,**D**). The hazard ratio (HR), 95% confidence interval (CI), and p-value for univariable Cox regressions are presented at the top of each graph. Time is provided in months. Subjects at risk are shown in a separate risk table under the graph.

There were no notable differences in patient characteristics between the MCL2, MCL3, and MCL6 cohorts except for the age at relapse (Supplemental Material, Table S1) and the number of previous treatments (Supplemental Material, Table S2). These differences are explained by the original trial’s inclusion criteria since a young age ≤65 years) and newly diagnosed disease were required in the MCL2 and MCL3 cohorts [20,21], whereas the MCL6 trial had no requirement regarding age and the number of prior treatments [22]. In the total cohort of all 65 patients, the circSCORE identified 14 patients as high risk (21.5%) and 51 patients as low risk (78.5%). This distribution was similar when considering the cohorts separately, with 20% and 22% of the patients assigned as having a high-risk circSCORE in MCL2/3 and MCL6, respectively (Supplemental Material, Table S1). To investigate the prognostic potential of circSCORE, survival analyses were first performed in the total cohort of all 65 patients. Intriguingly, the analyses showed significantly shorter PFS (hazard ratio (HR) 1.99, confidence intervals (CIs) 1.03–3.83, *p*-value 0.0407 (Figure 1A)) and OS (HR 2.29, CI 1.15–4.60, *p*-value 0.0192 (Figure 1B)) for the high-risk circSCORE group compared to the low-risk group.

### 3.2. Promising Prognostic Performance of circSCORE in Non-Nodal Tissue

Since lymph node biopsies are infrequently available in the R/R MCL setting, the prognostic potential of circSCORE in non-nodal tissue was explored independently in the MCL6 cohort (*n* = 45). The results displayed trends towards shorter PFS (HR 2.03, CI 0.91–4.53, *p*-value 0.0831 (Figure 1C)) and shorter OS (HR 2.09, CI 0.89–4.93 *p*-value 0.0911 (Figure 1D)) for patients with a high-risk circSCORE versus a low-risk circSCORE, although significance was not reached. To compare these results, survival analyses of the first relapse lymph node samples from patients in the MCL2/3 cohort were carried out as well. The trends observed in the nodal analyses (MCL6) were consistent with the results of circSCORE stratification in the MCL2/3 cohort and also displayed a trend of inferior outcome for patients in the circSCORE high-risk group for PFS (HR 2.14, CI 0.67–6.82, *p*-value 0.1971 (Supplemental Material, Figure S1A)) and OS (HR 3.13, CI 0.92–10.59, *p*-value 0.0669 (Supplemental Material, Figure S1B)). It should be noted that separate analyses were carried out on a reduced number of patients (*n* = 45 and *n* = 20 in MCL6 and MCL2/3, respectively) and, thus, the findings should be interpreted with appropriate caution.

### 3.3. The circSCORE Is Independently Prognostic for PFS

To explore whether the prognostic performance of circSCORE in the R/R setting was influenced by other known MCL high-risk features, multivariable Cox regression analyses were carried out. Full data on circSCORE and the high-risk features, MIPI, Ki67, and *TP53* mutation, all measured at relapse, were available for 49 of the 65 patients. Despite the reduced number of patients, the circSCORE retained a prognostic impact in the Cox regression models independently of the Ki67, MIPI, and *TP53* mutation status for PFS (HR 1.92, CI 1.13–3.26 *p*-value 0.0147), but not for OS (HR 1.31, CI 0.69–1.83, *p*-value 0.0625). MIPI retained prognostic impact for both PFS and OS, whereas Ki67 and *TP53* mutation status did not (Table 1). It should be noted that the combined MIPI-C was not included in these analyses to avoid the risk of overfitting when incorporating the same variables more than once [24].

In the total cohort of 65 patients, data on the circSCORE, Ki67, MIPI, and TP53 mutation were available for all 49 patients, and they were all measured at relapse. MIPI, circSCORE, and Ki67 were included on continuous scales to eliminate bias in the results due to the different number of risk groups that the scores assigned the patients into (high, intermediate, and low for MIPI versus high, and low for circSCORE and Ki67). However, since the *TP53* mutation status is a fixed categorical variable, it was included as so, and the *TP53* wild type was set as the reference. For the continuous scale of Ki67, hazard ratios were calculated per increment of 10% in accordance with the numerical scale range.

## 4. Discussion

This is the first study to examine the prognostic potential of the circRNA-based risk score, circSCORE, in R/R MCL. The results of this study highlight several interesting findings, including unfavorable outcomes for patients with R/R MCL in the high-risk circSCORE group and similar trends when explored in non-nodal tissue exclusively. Moreover, the prognostic impact of circSCORE remained significant for PFS when adjusting for the other high-risk features MIPI, *TP53,* and Ki67, even though the analysis included only 49 of 65 patients due to missing data. Also, the current results of the study imply the promising application of circSCORE in non-nodal tissues in R/R MCL disease, which has major advantages in a clinical setting, allowing the use of less invasive methods such as a blood test or a bone marrow biopsy. This is favorable for the patients, is less time-consuming, and reduces the risk of complications arising from lymph node resection. With the potential to overcome the need for a lymph node biopsy, circSCORE could offer a possible alternative to the immunohistochemical staining of the Ki67 proliferation marker, which is primarily suitable for lymph node tissue and should not be assessed in non-nodal tissues such as bone marrow, which contains admixed proliferating hematopoietic cells [25]. This is particularly challenging in the R/R MCL setting where lymph node biopsies are rarely available, and since Ki67-positive cells are not homogenously distributed in lymph node tissue, the selection of a representative area poses subjectivity challenges, even when following assessment guidelines [26]. The results of this study imply the promising compatibility of the circSCORE in various tissues, possibly overcoming the tissue-specific limitations of Ki67. Additionally, circRNA expression in MCL has previously demonstrated inverse association to Ki67 expression [5], supporting the theory that circRNAs are often less abundant in rapidly proliferating cells [13]. Furthermore, since circRNAs play key roles in oncogenic regulatory processes, including cell proliferation [27], circRNA-based biomarkers have the potential to serve as proliferation markers [28]. The circRNAs in the circSCORE, except circNCOA2, have all been indicated to play a role in cell proliferation in various cancer types, primarily through an interplay with miRNAs [29,30,31,32,33,34,35,36,37], yet none of the studies focused specifically on their role in lymphomagenesis nor resolved the spatial circRNA expression patterns within the tumor microenvironment, which is important in understanding the function of circRNAs as microRNA sponges [38]. Interestingly, a few recent studies have focused on other circRNAs and their regulatory properties in cell proliferation in some B-cell malignancies. This included circRIC8B, which was recently found to be upregulated in chronic lymphatic leukemia (CLL) and implied in sponging miR-999b-5p, increasing the levels of lipoprotein lipase (LDL), and thereby promoting lipid accumulation and cell proliferation in CLL cells [39]. In diffuse large B-cell lymphoma (DLBCL), the downregulation of circAPC implied sponging of miR-88 and activation of the oncogene, TET1, ultimately suppressing the Wnt/β-catenin pathways and promoting cell proliferation [40]. Also, in DLBCL, the overexpression of circOTUD7A was implied in sponging of miR-431-5p, thereby inhibiting FOXP1 suppression and promoting cell proliferation [41]. Still, the role of circRNAs in regulating cell proliferation and circRNA expression patterns in B-cell malignancies remains relatively unexplored. In addition to this, despite the fact that circSCORE might reflect a degree of cell proliferation, the possibility of the circSCORE replacing the Ki67 index as a surrogate for cell proliferation in MCL is still unclear, and further investigations in larger cohorts are warranted. However, in that case, circSCORE could offer a potential proliferation-associated marker free of inter- and intravariability linked to immunohistochemical methods, along with providing promising compatibility with various tissue samples. In relation to this, it should, therefore, be emphasized that the NanoString Technology on which the circSCORE is based has been shown to be an accurate, objective, and rapid method for RNA quantification. Other NanoString-based biomarkers for MCL have also recently been presented, including the MCL35 risk score, which was developed as a prognosticator in diagnostic lymph node samples [42], and the leukemic MCL16 (LMCL-16) score, which has been proposed as a diagnostic marker to distinguish MCL subtypes based on PB samples [43]. Recently, the MCL35 risk score was explored in R/R MCL, showing retained prognostic value when adjusted for MIPI and *TP53* mutation/deletion for PFS, but further validation is needed [44]. Both MCL35 and LMCL-16 have shown promising results, but these biomarkers require a high tumor load (≥60%) for reliable results and are based on mRNA, which is less stable compared to circRNA [45], and thus, more challenging to use in a clinical setting as it is susceptible to degradation during sample obtainment and the subsequent isolation of RNA. However, the ongoing proposal of NanoString-based biomarkers reflects the beginning of the implementation of NanoString in the clinic, including the PAM50 gene signature used in breast cancer and the Lymph2Cx assay used in diffuse large B-cell lymphoma.

### 4.1. Strengths, Limitations, and New Perspectives

Accurate prognostication at the time of relapse is a major challenge in MCL management, especially considering the highly heterogeneous nature of the disease and the increasing number of available treatment options. A fundamental strength of this study is its novelty in addressing the unexplored performance of circSCORE as a possible circRNA-based risk score in R/R MCL. Also, this study is the first to independently explore circSCORE in non-nodal tissue, as previous investigations of the circSCORE have exclusively included samples of both nodal- and non-nodal origin [5,6]. Considering the major clinical advantages of risk score applicable to various tissues, as discussed in the section above, the results of this study are some of the first to contribute to this unmet need. However, despite the intriguing results of the circSCORE in the R/R MCL setting, the limited number of patient samples remains a weakness in this study. Hence, this study was based on available samples from the Nordic MCL2, MCL3, and MCL6 patient cohorts and the necessity to combine these cohorts to ensure statistical power in the survival analyses, which created a degree of inhomogeneity in analyses in the total cohort. Therefore, separate survival analyses were also performed, respectively, for the MCL6 cohort (Figure 1C,D) and MCL2/3 cohort (Supplemental Material, Figure S1A,B); however, this inevitably led to statistical constraints associated with a reduced number of patients (*n* = 45 in MCL6 and *n* = 20 in MCL2/3) and fewer events. Furthermore, future analyses of the circSCORE should also focus on analyzing parried tissue samples for intra-patient comparisons. This would be highly valuable, providing the opportunity for an in-depth understanding of how the circRNA levels correspond in different tissue compartments of each patient. Additionally, considering that the patients in this study had all received intensive treatment prior to sample obtainment (Supplemental Material, Table S2), exploring the circRNA expression levels during the treatment course from baseline to relapse and from first to second relapse could provide valuable insights into the molecular alterations associated with disease progression in R/R MCL. This could also lead the way toward potential trajectories of targeted therapeutic interventions being explored in MCL. The important dynamics of circRNA in various tissue compartments [46] and the exploitation of circRNAs as biomarkers and targeted anticancer agents, in general, have been underscored in previous research [12,27,47,48] but not thoroughly investigated in lymphomagenesis. Due to the limitation of available sequential tissue samples across the course of the disease, the design of this study was not geared to perform such analyses. Nevertheless, the initial explorations of circSCORE application in R/R MCL presented in this study were promising overall, and if further validated, circSCORE could possibly be suitable for future clinical use.

## 5. Conclusions

This is the first study to investigate the circRNA-based risk score, circSCORE, in R/R MCL applied in both nodal and non-nodal tissues. The results of this study reveal a significantly inferior outcome for high-risk circSCORE R/R MCL patients in terms of both PFS and OS, as well as the promising use of circSCORE in non-nodal tissues. Furthermore, the prognostic impact of circSCORE for PFS is retained even after adjusting for Ki67, MIPI, and *TP53,*, despite the relatively small number of patients included in the study. However, these results warrant further investigation, preferably in larger cohorts including both nodal and non-nodal samples, to fully elucidate the future clinical utility of circSCORE.

## Figures and Tables

**Table 1:** **Prognostic impact of circSCORE, Ki67, MIPI, and *TP53* mutation status in adjusted Cox multivariable regression models.**

*n* = 49	Progression-Free Survival		Overall Survival	
Variables	HR	*p*-value and CI	HR	*p*-value and CI
circSCORE	1.92	0.0147 * 1.13–3.26	1.31	0.0625 0.69–1.83
Ki67	1.20	0.7892 0.98–1.13	1.07	0.2724 0.99–1.02
MIPI	2.32	0.0008 *** 1.42–3.78	1.92	0.0024 ** 1.26–2.94
*TP53* mutation	1.62	0.1944 0.78–3.38	1.46	0.3351 0.67–3.18

Abbreviations: *n*: number of patients, HR: hazards ratio, CI: 95% confidence interval. * *p*-value < 0.05, ** *p*-value < 0.01, *** *p*-value < 0.001.

## Data Availability

The data supporting the conclusions of this article will be made available by the authors upon request.

## Supplementary Materials

The following supporting information can be downloaded at: https://www.mdpi.com/article/10.3390/genes16060634/s1, Table S1: Patient and disease characteristics; Table S2: Treatment specifications; Figure S1: Kaplan Meier survival curves and Cox univariable regressions of PFS and OS stratified on circSCORE risk groups in the MCL2/3 cohort.
