# Supplementary material for: Exploring the Prognostic Potential of circSCORE in Patients with Relapsed/Refractory Mantle Cell Lymphoma
Source: Genes (Basel). 2025 May 25;16(6):634. doi: 10.3390/genes16060634 (PMC12193225; doi:10.3390/genes16060634)
Supplement: Supplementary file 1 [file genes-16-00634-s001.zip › Supplemental material.pdf]

Supplemental material (SM)

Exploring the prognostic potential of circSCORE in patients with relapsed/refractory mantle cell lymphoma

SM Tables:

Table S1: Patient and disease characteristics

Table S2: Treatment specifications

SM Figures:

Figure S1: Kaplan Meier survival curves and Cox univariable regressions of PFS and OS stratified on circSCORE risk groups in the MCL2/3 cohort

Table S1: Patient and disease characteristics

Variable	N	Level	MCL2/MCL3 n=20(%)	MCL6 n=45(%)	Total n=65(%)	P-value
Sex	65	Female	8(40.0)	12(26.7)	20(30.8)	0.43
		Male	12(60.0)	33(73.3)	45(69.2)	
Age at relapse	65	Median [min;max]	58.5 [46;68]	69 [48;80]	65 [45;80]	<0.001
ECOG	65	0-1	20(100.0)	41(91.1)	61(93.8)	0.41
		2-4	0(0.0)	4(8.9)	4(6.2)	
Tissue origin	65	LN	20(100)	0(0.0)	20(30.7)	-
		BM	0(0.0)	34(75.5)	34(52.3)	
		PB	0(0.0)	11(24.5)	11(17)	
BM involvement	64	No	11(55.0)	13(29.5)	24(37.4)	0.09
		Yes	9(45.0)	31(70.5)	40(62.2)	
WBC level	65	Normal	16(80.0)	38(84.4)	54(83.1)	0.93
		High	4(20.0)	7(15.6)	11(16.9)	
MIPI risk group	61	Low	9(56.2)	8(17.7)	17(27.9)	0.06
		Intermediate	5(31.2)	15(33.3)	20(32.8)	
		High	2(12.5)	22(48.9)	24(39.3)	
TP53 mutation	63	No	10(55.6)	34(75.6)	44(69.8)	0.21
		Yes	8(44.4)	11(24.4)	19(30.2)	
Ki67 index	55	<30%	3(23.1)	19(45.2)	22(40.0)	0.27
		≥30%	10(76.9)	23(54.8)	33(60.0)	
circSCORE	65	Low	16(80.0)	35(77.8)	51(78.5)	0.97
		High	4(20.0)	10(22.2)	14(21.5)	

P-values are calculated based on a Chi-squared test. No test is made for tissue origin since MCL2/3 only included lymph node and MCL6 only included non-nodal tissue. High WBC level is ≥10.7 × 10^9/L.

Table S2: Treatment specifications

Variable	N	Level	MCL2/MCL3	MCL6	Total	P-value
			n=20(%)	n=42(%)	n=62(%)	
Number of previous therapies	62	1	20(100.0)	11(26.2)	31(50.0)	<0.001
		2	0(0.0)	9(21.4)	9(14.5)	
		3	0(0.0)	9(21.4)	9(14.5)	
		≥4	0(0.0)	13(31.0)	13(21.0)	
			n=20(%)	n=31(%)	n=51(%)	
Type of relapse treatment	51	Chemoimmunotherapy	15(75.0)	31(100)	46(90.2)	0.09
		R-Benda	3	15	18	
		CHOP-based	3	7	10	
		Fludarabine-based	4	2	6	
		Cytarabine-based	1	9	10	
		Platin-based	1	0	1	
		Bortezomib-based	3	2	5	
		Immunomodulatory	4(20.0)	8(25.8)	12(23.5)	
		HDT+ASCT	0(0.0)	8(25.8)	8(15.7)	
		Radiotherapy	1(5.0)	6(19.4)	7(13.7)	
		BTKi	0(0.0)	4(12.9)	4(7.8)	
		Other	0(0.0)	12(38.7)	12(23.5)	
		Allogenic stem cell transplantation	0(0.0)	3(9.7)	3(5.9)	

P-values are calculated on a Fisher's exact test. Data on previous treatment was missing in 3 patients from the MCL6 cohort (n=42) and 31 patients had been treated with more than a first line treatment (n=31). Type of relapse treatment is registered when given to the patient at any time during the relapse course. HDT+ASCT was given first line to all patients in MCL2/3, whereas 13 patients included in MCL6 had received ASCT first line, and 8 as part of relapse treatment. Immunomodulatory therapy includes either Rituximab monotherapy, Lenalidomide or Campath. CHOP-based regmien includes the R-EPOCH, R-CHOP or R-CHOEP. Fludarabine-based includes either Fludarabine-Cyclophosphamide or Rituximab-Fludarabine-Cyclophosphamide. Platin-based includes Rituximab-Ifosfamide-Carboplatin-Etoposid. Bortezomib-based includes either Cisplatin-Vincrisine-Dacarbazine or Rituximab-Bortezomib. BTKi includes Lenalidomid and Ibrutinib. The category 'other' consist of either Corphase, Carmustine, Leukuran or other-non-specified experimental treatment. Abbreviations: BTKi: Bruton's Tyrosine Kinase inhibitor, R-Benda: Rituximab-Bendamustine, R-EPOCH/R-CHOP/R-CHOEP: Rituximab, Etoposide, Vincristine, Prednisone, Cyclophosphamide, and Doxorubicin, HDT: high dose therapy, ASCT: autologous stem cell therapy, RT: radiotherapy.

Figure S1: Kaplan Meier survival curves and Cox univariable regressions of PFS and OS stratified on circSCORE risk groups in the MCL2/3 cohort

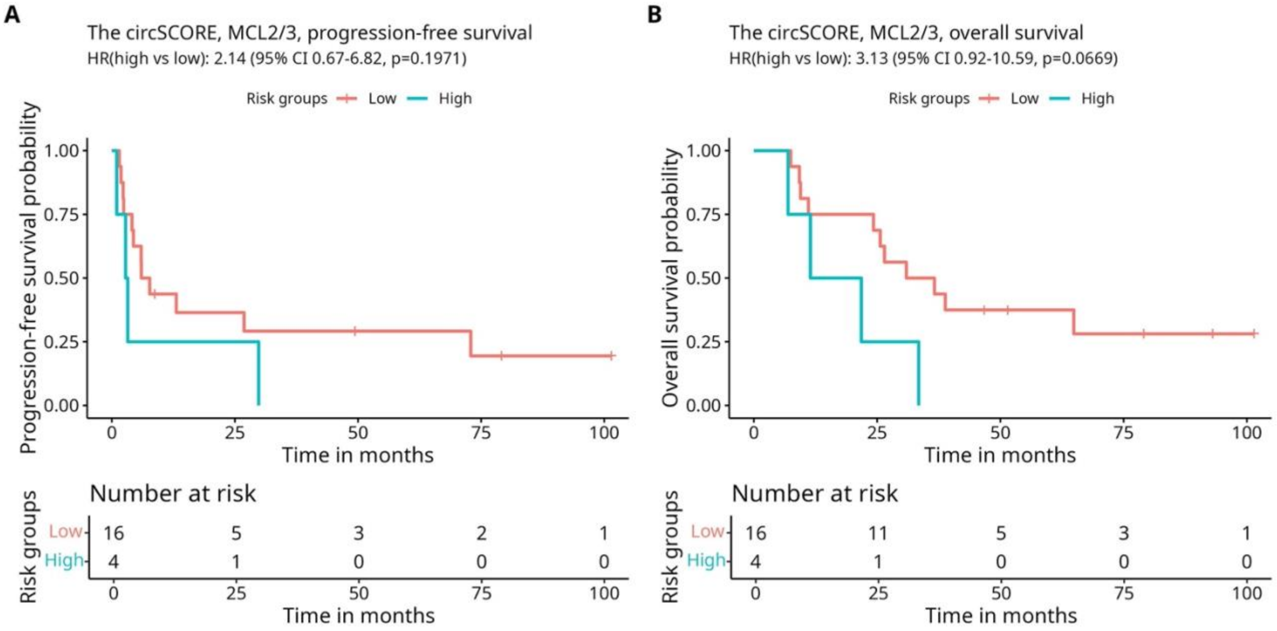

Kaplan-Meier survival curves and univariable Cox regressions performed using the 20 available first relapse lymph node samples from patients in the MCL2 and MCL3 (MCL2/3) cohorts. Patients are stratified in circSCORE high- and low-risk groups, and endpoints are progression free survival (PFS) (A) and overall survival (OS) (B). Hazard ratio (HR), 95% confidence interval (CI) and p-value for univariable Cox regressions are presented at the top of each graph. Time is provided in months. Subjects at risk are shown in the separate risk table under the graph.
